# A Solitary Abscessed Hydatid Cyst of the Buttock: A Rare Case

**DOI:** 10.7759/cureus.38823

**Published:** 2023-05-10

**Authors:** Mohamed Chablou, Ahmed Id M'barek, Rania Chacha, Abdelali Merhom, Mohamed Tahi

**Affiliations:** 1 General Surgery, Mohammed VI University Hospital Center, Oujda, MAR; 2 Pulmonary Medicine, Moulay Ali Cherif Regional Hospital, Errachidia, MAR; 3 Oncology, Mohammed VI University Hospital Center, Oujda, MAR; 4 General Surgery, Ibn Rochd University Hospital Center, Casablanca, MAR; 5 General Surgery, Faculty of Medicine and Pharmacy, Hassan II University of Casablanca, Casablanca, MAR

**Keywords:** hydatid cyst abscess, cystic echinococcosis, infected hydatid cyst, buttock hydatid cyst, cyst hydatid

## Abstract

The hydatid cyst is a cosmopolitan parasitic infection caused by tapeworms of the genus *Echinococcus* and is a major public health problem in developing countries. Solitary hydatid cysts located in the buttocks are very rare, and the unusual location of the cyst can aid in the differential diagnosis of subcutaneous masses in this area, particularly in endemic areas. In this report, we present the case of a 39-year-old man who was admitted to the emergency department with a painful, abscessed cyst in the buttock region. The cyst was completely excised, and histopathological examination confirmed the diagnosis of a hydatid cyst. Further investigations did not reveal any other locations. Although the buttock region is an extremely rare site of infection for a hydatid cyst, it should be considered in cystic lesions, especially in endemic areas.

## Introduction

Human echinococcosis is a zoonotic disease caused by parasites of the *Echinococcus* genus. In developing countries such as Morocco, cystic echinococcosis or hydatidosis is a significant public health concern in areas where livestock are raised [1]. This cosmopolitan anthropozoonosis is caused by the development of the *Echinococcus granulosus* larva in humans. The primary host is the dog, and humans become accidental intermediate hosts by ingesting parasite eggs from contaminated food or through direct contact with infected dogs [2]. The liver (60%-70%) and lungs (5%-27%) are the most commonly affected organs, and the occurrence of a primary and isolated hydatid cyst in the subcutaneous tissues of the buttocks is uncommon and rare, even in highly endemic areas [3,4]. Preoperative diagnosis of a hydatid cyst abscess can be challenging and may be confused with other differential diagnoses. We report a rare case of a 39-year-old man who presented to the emergency department with an abscessed mass in his right buttock. The diagnosis of hydatid cyst was made postoperatively after histopathological examination.

## Case presentation

**Figure 1:**
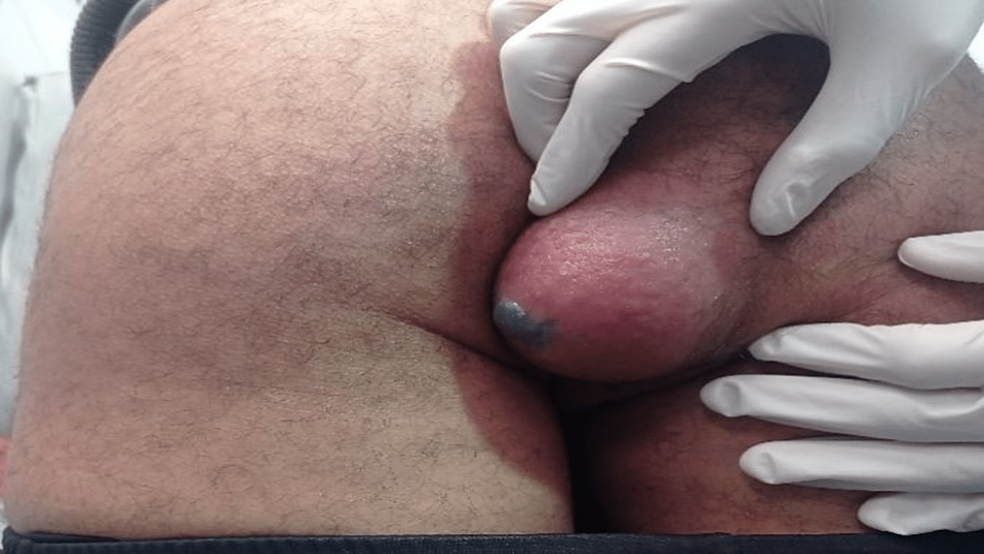
Abscessed buttock mass

The patient was a 39-year-old man who worked as a farmer and had a history of contact with dogs. He presented with a small right buttock mass that had been slowly growing for the past six months, which was painless and asymptomatic. A physical examination revealed a firm, tender, and poorly mobile mass with inflammatory signs, while the proctological examination was normal (Figure 1).

**Figure 2:**
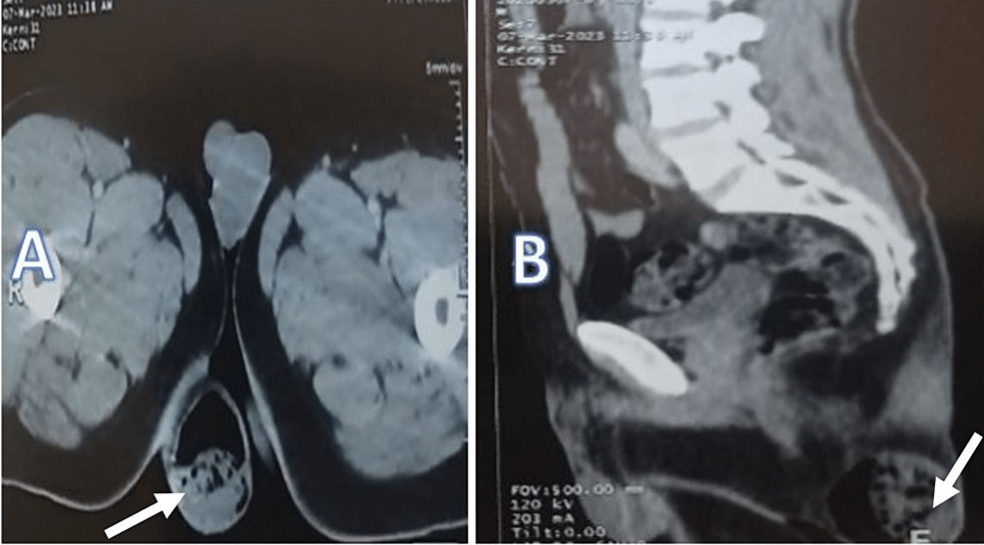
Computed tomography scan showing buttock mass containing air bubbles (white arrow) (A) Sectional axial image; (B) sagittal section

The patient had a temperature of 38.5°C. Laboratory tests showed a white blood cell count of 16,000/mm^3^ and a C-reactive protein level of 90 mg/L. A non-contrast pelvic CT scan showed a buttock mass containing air bubbles without extension to adjacent structures, while the chest radiograph was normal (Figure 2).

**Figure 3:**
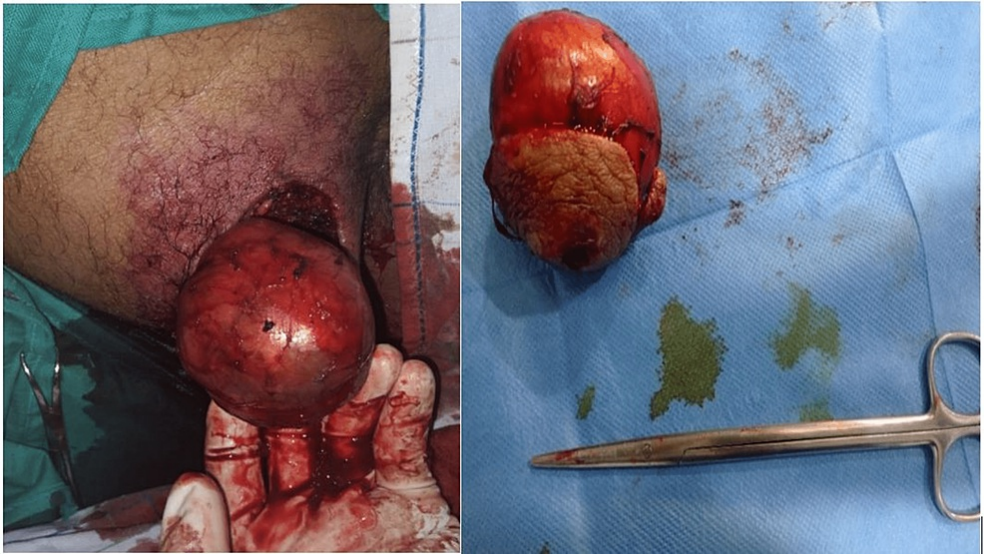
Surgical images showing the complete resection of the buttock mass

The patient underwent surgical intervention under spinal anesthesia, and a complete resection of the mass was performed (Figure 3).

**Figure 4:**
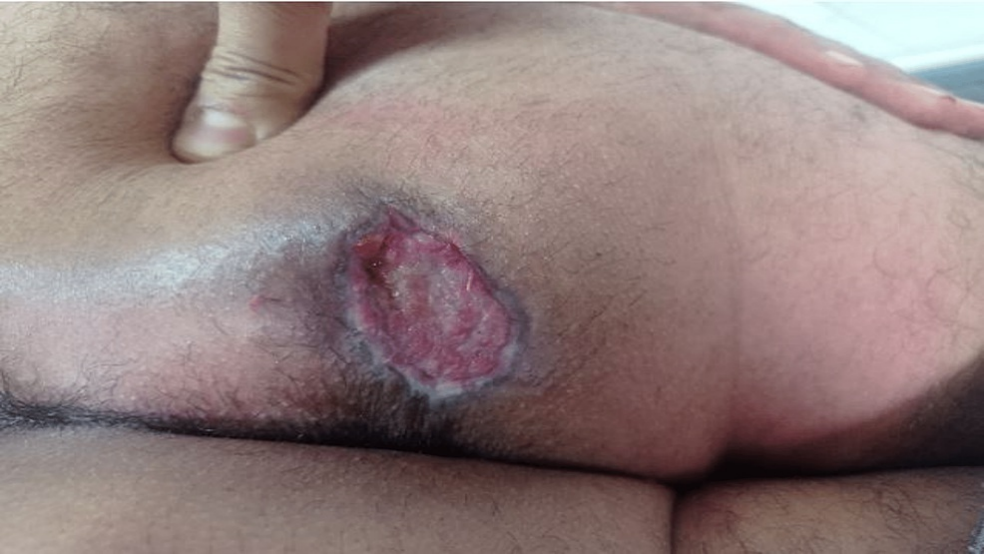
Image showing a good wound healing on follow-up

The histopathological examination of the operative specimen confirmed the diagnosis of an infected hydatid cyst. The patient was discharged on day 2 with good wound healing on follow-up (Figure 4). A postoperative thoraco-abdominal-pelvic CT scan did not show any hydatid cyst localization.

## Discussion

Hydatidosis is an endemic disease that affects many countries in Africa, the Middle East, the Mediterranean, and South America, which are developing livestock countries [1]. It is a cosmopolitan anthropozoonosis that represents a real global health problem, caused by the development of the larval form of *Echinococcus granulosis* in humans. The dog is the definitive host, and human infection occurs accidentally by ingestion of food contaminated with the parasite's eggs or by direct contact with a sick dog. The ingested parasite eggs penetrate the intestinal wall and use the portal system to spread throughout the body. This explains the frequency of liver involvement (60%-70% of cases) and lung involvement (5%-27% of cases) [2-4]. Unusual localizations have been reported, including bone (1%-3%), pleura or peritoneum (4%-7%), spleen and kidney (2%-5%), brain (1%-5%), heart (0.5%-2% of hydatidosis localizations), and rarely the thyroid, pancreas, ovaries, joints, subcutaneous soft tissues, and muscles [5]. The majority of reported cases of subcutaneous hydatid cysts are located in the thigh (27%), hypogastric region, thorax, and head and neck region [2]. Gluteal localization is extremely rare (9% of subcutaneous hydatidosis cases), which explains the particularity of our observation [6].

Our patient, who had a history of contact with dogs, presented with a painful, warm, red, and "orange peel" appearance on the skin overlying an isolated buttock mass. The proctological examination was crucial in this case to rule out a perianal abscess, given the location of the mass near the anal margin, and no anal fistula or fissure was found. Laboratory tests confirmed the presence of an infection, and empirical antibiotic therapy was initiated. Hydatid serology was not requested given the urgency of the situation and the lack of diagnostic orientation; besides, it is not very sensitive for localizations in soft tissues, given the frequency of false negatives. However, this serology is a means of monitoring treatment when positive [7]. In our case, the diagnosis of an abscessed hydatid cyst was made postoperatively because the clinical presentation was poor and nonspecific, and the urgent context required rapid surgery. Imaging was used to investigate the local-regional relationships of the mass. We chose to perform a non-contrast perineal CT scan, which confirmed the subcutaneous abscessed cystic mass without extension to neighboring organs, but it did not determine the etiological diagnosis. Outside the emergency context, magnetic resonance imaging remains the diagnostic method of choice in hydatidosis of soft tissues when other imaging methods are inconclusive. It allows a detailed study of the wall, showing a peripheral rim with relative hypointensity on T2, as well as the cystic content, including daughter cysts and membranous structures, and allows for better analysis of the local-regional relationships essential for surgical planning [8-9].

Isolated subcutaneous localization of hydatid cysts is rare, representing only 2.3% even in endemic areas, making etiological diagnosis sometimes difficult [2]. Treatment is mainly surgical since it is the only radical treatment that confirms the diagnosis and ensures complete healing. Our patient underwent surgery under spinal anesthesia with complete resection of the abscessed cyst and its pericyst.

## Conclusions

The solitary hydatid cyst in the subcutaneous tissue is a rare occurrence, and its location in the buttocks is exceptional. In cases of complicated hydatid cysts in the buttocks with poor and nonspecific clinical presentation, surgery represents the only radical treatment, and histopathological examination confirms the diagnosis. Imaging methods such as ultrasound and CT scan can provide diagnostic guidance, but MRI remains the most effective examination. Hydatid serology has limited sensitivity. Despite the rarity of subcutaneous hydatidosis, it should always be considered in individuals living in endemic areas with any mass in the subcutaneous tissues, and additional tests should be requested to confirm the diagnosis and prevent therapeutic errors. The eradication of this condition relies on prophylaxis through health education of populations, monitoring of animal slaughter, and treatment of domestic dogs.
